# Analysis of the effect of PM10 on hand, foot and mouth disease in a basin terrain city

**DOI:** 10.1038/s41598-018-35814-5

**Published:** 2019-03-01

**Authors:** Fei Yin, Yue Ma, Xing Zhao, Qiang Lv, Yaqiong Liu, Xiaosong Li, Tao Zhang

**Affiliations:** 1 West China School of Public Health, Sichuan University, Chengdu, Sichuan People’s Republic of China; 2 Sichuan Center for Disease Control and Prevention, Chengdu, Sichuan People’s Republic of China

## Abstract

Hand, foot, and mouth disease (HFMD) is a common childhood infection that causes a substantial disease burden in the Asia-Pacific region. Various climate variables, such as humidity and temperature, have been associated with HFMD. However, few studies have assessed the impact of PM_10_ on childhood HFMD. This study investigated the association between PM_10_ and HFMD. We fitted a standard distributed lag non-linear model to investigate the temporal lagged relationship between PM_10_ and HFMD, and then further assessed whether this relationship varied by gender and pathogen. Between 2011 and 2015, a total of 122,564 HFMD cases under 15 years of age were reported in Chengdu. The PM_10_-HFMD associations were shown to be non-linear in all subgroups, with the peak at 101–218 μg/m^3^. Male children were more sensitive to PM_10_ effects. For pathogen-specific relative risks, we found that the risk estimates were generally higher in cases of CVA16 infection. Our study provides evidence that PM_10_ increases the risk of HFMD. Authorities and parents should be fully aware of the impact of PM_10_ on childhood HFMD. Furthermore, appropriate protective measures should be taken to reduce risks.

## Introduction

Hand, foot, and mouth disease (HFMD) is a common childhood infection that usually affects infants and young children^1^. HFMD is typically characterized by fever, mouth ulcers, and ulcerations on the hands, legs, or buttocks and mouth^2^. It is predominantly caused by coxsackievirus A16 (CVA16) and enterovirus 71 (EV71). The infection is usually mild and self-limiting; however, some HFMD patients may rapidly develop serious health consequences due to neurological and systemic complications, especially those infected with EV71^3^.

During the last few decades, many large outbreaks of HFMD have occurred in the Asia-Pacific region^4–7^. In China, HFMD is the most commonly reported infectious disease among the nationally notifiable diseases in recent years. There have been millions of reported cases and hundreds of reported deaths per year since 2008. From 2011 to 2015, more than 10.39 million cases were reported in China, including 1958 fatal cases. Given its serious threat to public health, a crucial issue in the prevention of HFMD is to identify environmental factors that may have significant impacts on the disease.

It has been well documented that climate factors often play significant roles in the transmission of HFMD^5,8–12^. Humidity and temperature are the most frequently reported climate factors. But, until now, little has been known about the impact of particulate matter under 10 microns (PM_10_) on HFMD. The impact of air pollution on respiratory diseases has been demonstrated by many epidemiological studies^13,14^, but very few studies have documented the link between PM_10_ and childhood HFMD. There are only two published papers addressing the effect of PM_10_ on HFMD, and their findings are inconsistent. Huang *et al*. demonstrated that there was no statistically significant relationship between PM_10_ and HFMD^15^, while Luo *et al*. reported a negative correlation between PM_10_ and HFMD^16^. Furthermore, no studies have assessed whether the PM_10_-HFMD relationship varies by gender or pathogen. The present study aimed to address these gaps.

Chengdu is located in Southwest China, which is the provincial capital of Sichuan Province. It is one of the most populous cities in China and is located in the west of the Sichuan Basin. Due to the surrounding mountains, winds enter the basin with an extremely reduced speed. The winds are relatively calm in Chengdu, with an average wind speed of 1.07 m/s. The basin terrain makes it difficult to disperse air pollutants^17^. In 2013, for instance, there were only 139 days when the air quality in Chengdu met the national air quality standard of 70 μg/m^3^ (24-hour average) for PM_10_^18^. This number (139 days) was lower than those in most of the other big cities in China, such as Tianjin (145 days), Beijing (167 days), Guangzhou (259 days), and Shanghai (246 days)^18^.

In this study, we investigated the relationship between PM_10_ and HFMD, and then further assessed whether this relationship varied by gender and pathogen. Specifically, we fitted a standard distributed lag non-linear model to examine the nonlinear lagged effects of daily PM_10_ on HFMD incidence. A better understanding might be attained for the association between PM_10_ and HFMD by adopting a more sophisticated model.

## Results

From January 1, 2011 to December 31, 2015, a total of 122,564 HFMD cases under 15 years of age were reported in Chengdu, of which 5,000 (4.08%) were laboratory confirmed. About 97% of HFMD cases occurred in children aged 0–5 years. Of 122,564 HFMD cases, 72,401 were males and 50,163 were females (male-to-female sex ratio: 1.44). Among the laboratory confirmed cases, 1,418 (28.36%) were associated with EV71, and 971 (19.42%) were associated with CVA16. Table 1 shows the descriptive statistics for meteorological variables, PM_10_, and HFMD cases. On average, there were 67.12 daily HFMD cases during the study period. The mean values of daily PM_10_ concentration, relative humidity, and mean temperature were 119.51 μg/m^3^, 78.47%, and 16.33 °C, respectively. The mean value of daily SO_2_ and NO_2_ were 25.59 μg/m^3^ and 55.21 μg/m^3^, respectively. The daily mean PM_10_ concentration significantly exceeded the values recommended by the national air quality standards and the World Health Organization (WHO).

**Table 1:** Descriptions of daily weather conditions and HFMD count in Chengdu.

	Mean	SD	Min.	Median	Max.
**Cases**					
HFMD cases	67.12	46.59	0	58.00	303
HFMD cases in males	39.65	27.64	0	34.00	175
HFMD cases in females	27.47	19.76	0	23.00	128
EV71-related cases	0.78	1.30	0	0.00	11
CVA16-related cases	0.53	1.01	0	0.00	9
**Meteorological variables and Air pollutants**					
Mean temperature (°C)	16.33	7.41	−0.40	17.40	29.20
Relative humidity (%)	78.47	8.58	32.00	79.00	97.00
PM_10_ (μg/m^3^)	119.51	74.02	16.00	102.00	862.00
NO_2_ (μg/m^3^)	55.21	18.61	15.00	52.00	144.00
SO_2_ (μg/m^3^)	25.59	14.51	4.00	23.00	96.00

**Figure 1:**
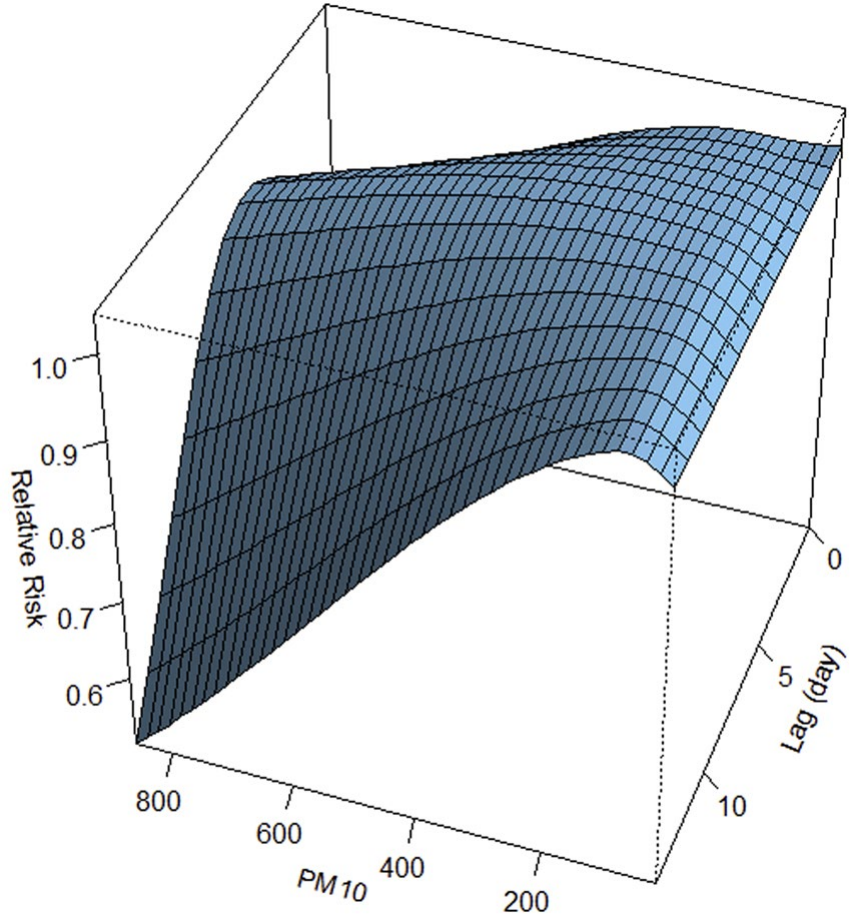
3D plot of the association between PM_10_ and HFMD over 14 days.

The 3D plot displays the association between PM_10_ and HFMD incidence during 14 lag days (Fig. 1). Overall, there was a non-linear association between PM_10_ and HFMD incidence. In addition, the results suggested that the association between HFMD and Pm_10_ might have a different lag structure. For instance, low PM_10_ (50 μg/m^3^) had a minimum relative risk (RR) at lag day 0 (the current day) and arrived the peak at lag day 9, and declined slowly on the following days. High PM_10_ (300 μg/m^3^) had a maximum RR at lag day 5, which declined rapidly on the following days.

**Figure 2:**
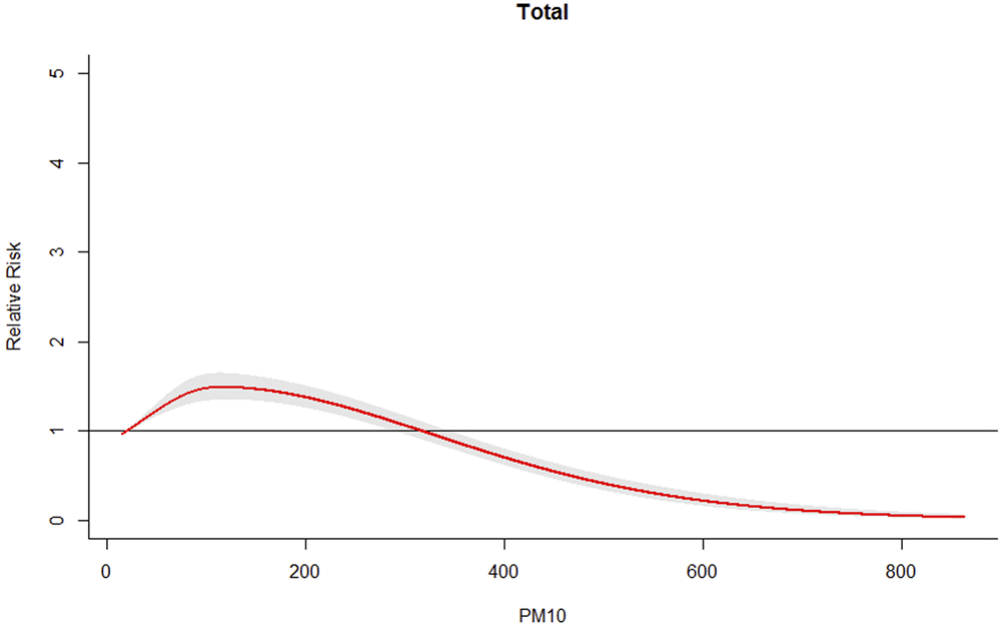
Cumulative RRs of PM_10_ for total HFMD cases.

Figure 2 illustrates the cumulative impact of PM_10_ on HFMD over the 14-day period. The results indicated that PM_10_ was statistically associated with HFMD. We found that the PM_10_-HFMD relationship exists as an approximately inverted V-shape curve. The cumulative RR increased with PM_10_. The curve peaked at 118 μg/m^3^, and then the curve began to decline.

**Figure 3:**
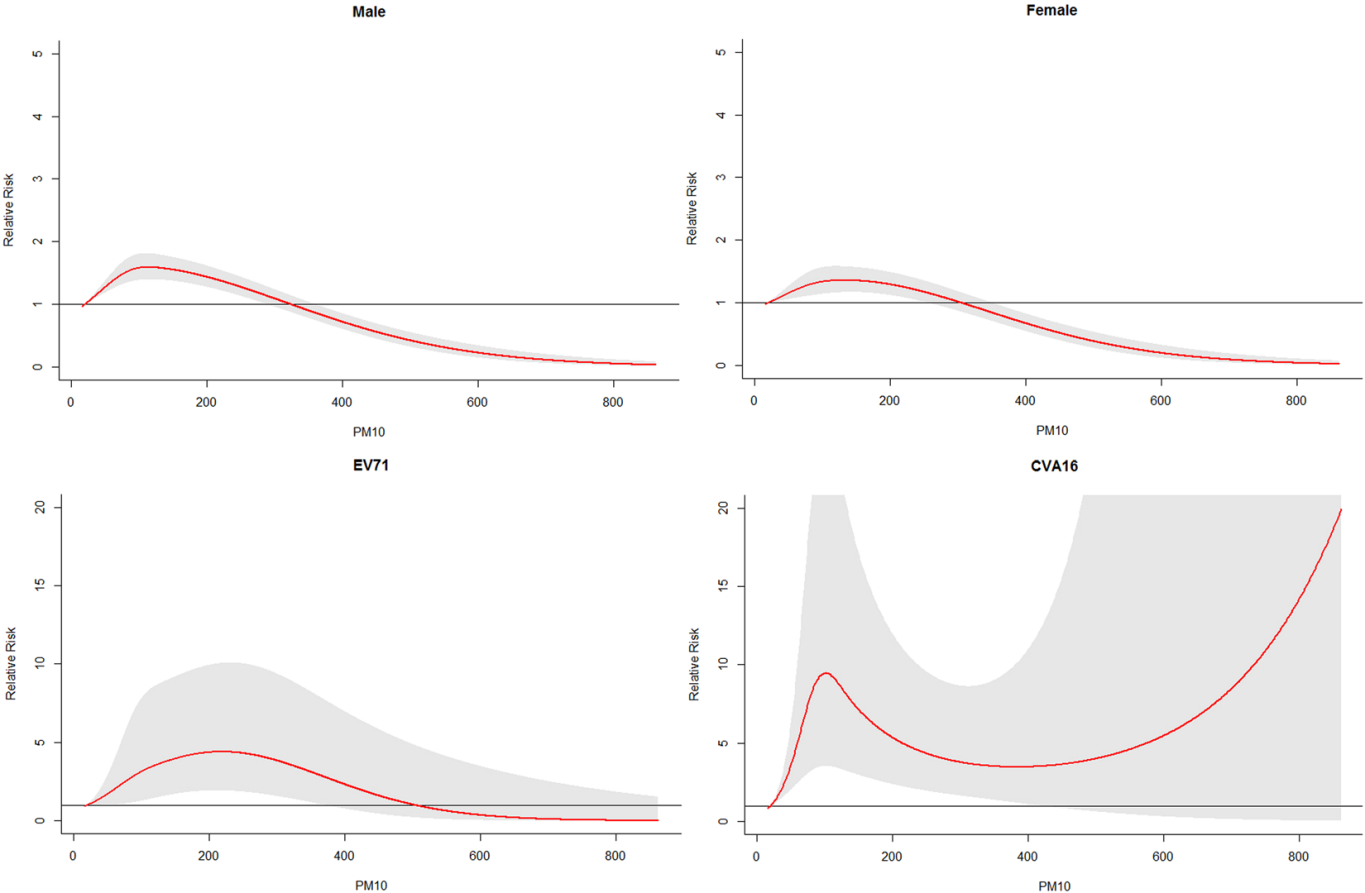
Cumulative RRs of PM_10_ for HFMD cases in different subgroups.

Figure 3 presents the cumulative RRs of PM_10_ on HFMD incidence over 14 days in different subgroups. For different gender groups, the exposure-response curves followed similar patterns. The cumulative RR reached a peak at 113 μg/m^3^ for male children and 131 μg/m^3^ for female children.

The cumulative effects of PM_10_ on different pathogen groups are also shown in Fig. 3. We found that the relationship between PM_10_ and HFMD varied across pathogen groups. For EV71-related cases, the exposure-response curve reached a peak at 218 μg/m^3^, and then the curve began to decline. For CVA16-related cases, the exposure-response curve reached a small peak at 101 μg/m^3^, and then the curve started to decrease. Again, the curve increased when PM_10_ was above 383 μg/m^3^.

The relative risks of different PM_10_ values along the lags for total, gender-, and pathogen-specific HFMD cases are displayed in Table 2. For gender-specific RRs, the risk estimates were generally higher in male children. For female children, the highest RR was 1.36 (95% CI: 1.17–1.58) at lag 0–14. For male children, the highest RR was 1.59 (95% CI: 1.40–1.81) at lag 0–14. When comparing risks for different pathogen groups, we found that the risk estimates were generally higher in CVA16-related cases. For CVA16-related cases, the highest RR was 9.46 (95% CI: 3.54–25.25) at lag 0–14, while for EV71-related cases, the highest RR was 4.40 (95% CI: 1.93–10.06) at lag 0–14 when PM_10_ was 101 μg/m^3^.

**Table 2:** RRs of different PM_10_ values for HFMD cases in different subgroups.

	PM_10_ (μg/m^3^)^a^	RR(95%CI)		
		Lag0	Lag7	Lag 0–14 (overall effect)
total	70	0.99(0.98,1.01)	1.03(1.02,1.04)	1.36(1.25,1.48)
	149	0.99(0.98,1.01)	1.04(1.03,1.05)	1.47(1.34,1.61)
	118^b^	0.99(0.98,1.01)	1.04(1.03,1.05)	1.49(1.35,1.65)
male	70	1.00(0.98,1.02)	1.03(1.02,1.04)	1.45(1.30,1.62)
	149	1.01(0.99,1.03)	1.04(1.03,1.06)	1.55(1.38,1.75)
	113^b^	1.01(0.98,1.03)	1.04(1.03,1.06)	1.59(1.40,1.81)
female	70	0.98(0.96,1.00)	1.02(1.01,1.04)	1.25(1.09,1.43)
	149	0.98(0.95,1.00)	1.04(1.02,1.06)	1.36 (1.17,1.57)
	131^b^	0.98(0.95,1.00)	1.04(1.02,1.05)	1.36(1.17,1.58)
EV71	70	0.91(0.81,1.03)	1.19(1.10,1.29)	2.31(1.08,4.98)
	149	0.92(0.79,1.07)	1.24(1.13,1.36)	3.95(1.70,9.17)
	218^c^	0.94(1.80,1.11)	1.12(1.12,1.35)	4.40(1.93,10.06)
CV-A16	70	1.12(0.96,1.30)	1.11 (1.02,1.21)	6.65(2.89,15.31)
	149	1.10(0.93,1.30)	1.15(1.04,1.26)	7.20(2.99,17.29)
	101^d^	1.14(0.95,1.36)	1.14(1.03,1.27)	9.46(3.54,25.25)

^a^70 μg/m^3^ and 149 μg/m^3^ represent the 25th percentile and 75th percentile of PM_10_, respectively;

^b^Peak value of PM_10_ for total children and gender-specific HFMD cases, respectively.

^c^218 μg/m^3^ represents the peak value of PM_10_ for EV71-related cases.

^d^101 μg/m^3^ represents the first small peak value of PM_10_ for CVA16-related cases.

In the sensitivity analyses, similar trends were observed when the degrees of freedom for weather factors and air pollutants were changed between 3 and 6.

## Discussion

Ambient air pollution has become one of the world’s biggest public health issues because of its profound impacts on the vast majority of the world’s population. The present research quantified the effect of PM_10_ on childhood HFMD, using Chengdu as the research area. The daily mean PM_10_ concentration in Chengdu significantly exceeded the values recommended by the national air quality standards and WHO. The current study has several strengths. To our knowledge, this is the first research to investigate the association between PM_10_ and childhood HFMD in a basin city, and to further evaluate gender- and pathogen-specific effects.

In this study, we fitted a standard distributed lag non-linear model to investigate the temporal lagged relationship between PM_10_ and HFMD. Our findings showed that an association exists between PM_10_ and HFMD. The exact mechanisms are not known; however, one possible explanation is that viruses may attach to the tiny particles in the air, thus facilitating the transmission of HFMD. In addition, we found an inverted V-shape pattern for the exposure-response curve. One possible reason is that the susceptible population might prefer staying at home or taking measures (e.g., wearing masks) to protect themselves on high air pollution days.

EV71 and CVA16 have been widely considered to be the predominant pathogens causing outbreaks of HFMD worldwide^19,20^. However, no studies have quantified the effects of PM_10_ on HFMD among different pathogen groups. We found that the PM_10_-HFMD relationship differed between EV71 infection and CVA16 infection. Further research is needed to examine the pathogen-specific effects of meteorological factors and air pollutants on HFMD incidence.

The results of our study also suggest that the relationship between PM_10_ and HFMD varies by gender. The results indicated that males appeared to be more sensitive to PM_10_ effects. The results of our study are consistent with the previous study. A recent study also indicated that male children owned more prominent cumulative effects than female children^21^. Compared to female children, male children are generally more active and more likely to play outside, which increases their chances of being exposed to air pollutants and enteroviruses.

The pattern of the relationship between PM_10_ and HFMD risk has not been consistent among studies. Our study suggests an inverted V-shape pattern for the exposure-response curve over 14 days. Huang *et al*. found no statistically significant relationship between PM_10_ and HFMD in Ningbo city from 2012 to 2014^15^, while Luo *et al*. reported a negative correlation between PM_10_ and HFMD in Yuexiu district from 2010 to 2011^16^. These discrepancies could be attributed to various reasons, such as heterogeneity of analytical methods and different climatic and geographic conditions among the cities. For instance, Luo *et al*. used correlation analysis in their research without considering the non-linear and lagged effects of PM_10_ on HFMD. In addition, Luo *et al*. examined the effect of PM_10_ on HFMD at a monthly scale, which cannot provide detailed information. Furthermore, the mean value of the PM_10_ concentration was 58.2 μg/m^3^ in Yuexiu District^16^, which was much lower than the mean value in Chengdu City (119.51 μg/m^3^). More studies are needed in different regions, especially in heavily polluted cities.

The pattern of the relationship between PM_10_ and HFMD risk has not been consistent among studies. Our study suggests an inverted V-shape pattern for the exposure-response curve over 14 days. Huang *et al*. found no statistically significant relationship between PM_10_ and HFMD (from 2012 to 2014) and a certain relationship between PM_10_ and female HFMD cases in Ningbo city (from 2012 to 2016)^15,22^. Luo *et al*. reported a negative correlation between PM_10_ and HFMD in Yuexiu district from 2010 to 2011^16^. A recent study in Guilin city provided a clue that a high PM_2.5_ level increased the risk of HFMD, though it was not statistical significant^21^. These discrepancies could be attributed to various reasons, such as heterogeneity of analytical methods and different climatic and geographic conditions among the cities. For instance, Luo *et al*. used correlation analysis in their research, while Huang *et al*., Yu *et al*., and our study adopted DLNM in the studies. In addition, Luo *et al*. examined the effect of PM_10_ on HFMD at a monthly scale, while Huang *et al*., Yu *et al*., and our study examined the effect at a daily scale. Furthermore, the degree of air pollution varies among the cities. For instance, the mean values of the PM_10_ concentration were 58.2 μg/m^3^ and 72.0 μg/m^3^ in Yuexiu District and Guilin city, respectively. These values were much lower than the mean value in Chengdu City (119.51 μg/m^3^). More studies are needed in different regions, especially in heavily polluted cities.

Some limitations of the current study should be acknowledged. First, there are complicated associations among air pollutants. However, only SO_2_ and NO_2_ were adjusted in the distributed lag nonlinear model (DLNM) since the data of other air pollutants (such as CO and O_3_) were only available since 2013. Those air pollutants will be examined once a longer time series is obtained. Second, the present research focused on the effect of PM_10_ on HFMD. Multi-pollutant models might have a great influence on the robustness of parameter estimation, which might results in the difficulty and limitations of result interpretation^23^. Therefore, when estimating the health impacts of particulate matter, the results of the single-pollutant models were more often reported and may be more representative^23^. In addition, potential interactions may exist between meteorological factors and air pollutants. Further research is required to investigate the interaction effects of meteorological factors and air pollutants on HFMD incidence.

In conclusion, our study provides evidence that PM_10_ could increase the risk of HFMD. Authorities and parents should be fully aware of the impact of PM_10_ on childhood HFMD. Appropriate protective strategies should be taken to reduce the risks. Furthermore, different PM_10_–HFMD relationships among different subgroups would be helpful in optimizing prevention and control strategies.

## Materials and Methods

### Data sources

In 2004, a nationwide notifiable infectious diseases reporting information system (NIDRIS) was established in China. In 2008, HFMD was listed as a class C notifiable infectious disease. All of the HFMD cases were required to be reported to the NIDRIS within 24 hours after diagnosis. The diagnosis of HFMD cases was based on the national clinic guide published by the Chinese Ministry of Health^24^. Daily cases of HFMD in Chengdu during 2011–2015 were obtained from NIDRIS. According to the preliminary analysis, 99.60% of HFMD cases occurred among children aged 0–14 years during the study period. So HFMD cases under the age of 15 years were chosen in the present study.

Daily data of air pollutants from 2011 to 2015 were collected from the Sichuan Environmental Monitoring Center, including PM_10_, SO_2,_ and NO_2_. Meteorological data were obtained on a daily basis from the China Meteorological Data Sharing Service System.

### Statistical analyses

In the present study, a time-series regression based on a generalized linear model with a poisson family was used to examine the effect of PM_10_ on HFMD incidence. In this regression, the association with PM_10_ was specified with a standard distributed lag nonlinear model (DLNM)^25,26^, which can model simultaneously non-linear and lagged effects. The model is shown below:

$$\begin{array}{lll}\mathrm{log}\,[E({Y}_{t})] & = & \mathrm{int}\,ercep+cb(p{m}_{10})+ns({\rm{weather}}\,{\rm{variables}})+ns({\rm{air}}\,{\rm{pollutants}})\\  &  & +{\beta }_{1}Trend+{\beta }_{2}Holiday+{\beta }_{3}Dow,\end{array}$$

where t indicates study days, ${Y}_{t}$ denotes the daily number of HFMD cases, and cb(pm_10_) means the cross-basis function for the space of the predictor and the lag dimension of daily PM_10_. We selected a cross-basis composed of a natural cubic spline for the exposure response, and a natural cubic spline for the lag response. The Akaike Information Criterion was used to evaluate the choice of degrees of freedom (df)^25^. The lag period was extended to 14 days to investigate the potential lag associations based on the incubation period of HFMD infections (usually around 3–5 days)^24^ and previous studies^11,12^. ns(weather variables) represents natural cubic spline fits to relative humidity and mean temperature, both with three df. ns(air pollutants) represents natural cubic spline fits to SO_2_ and NO_2_, both with three df. Trend represents a variable of the calendar month and year, which is used to control for long-term trends and seasonal effects. Dow represents the day of the week. Holiday is a binary variable indicating whether day *t* was a public holiday. ${\beta }_{1}$, ${\beta }_{2}$, and ${\beta }_{3}$ represent the regression coefficients.

Stratified analyses were conducted by gender (female and male) and pathogen group (CVA16 and EV71). We performed sensitivity analyses to evaluate the robustness of the results: changing the df (3–6) for climate factors and air pollutants. R packages, including “spline” and “dlnm”, were adopted to create the model.

### Ethics approval

In 2008, HFMD was listed as a class C notifiable infectious disease. The present study was based on official HFMD surveillance data in Sichuan Province. No confidential information was included because analyses were performed at the aggregate level. The research protocol was approved by the institutional review board of the School of Public Health, Sichuan University. In the current study, all of the methods were conducted in accordance with the approved research protocol. Since all of the patients’ records were anonymized and no individual information can be identified, informed consent was not required.

## Data Availability

The datasets generated and/or analyzed during the current study are available from the corresponding author on reasonable request.

## References

[1] Xing W (2014). Hand, foot, and mouth disease in China, 2008–12: an epidemiological study. Lancet Infect Dis.

[2] Jiang M (2012). Autopsy findings in children with hand, foot, and mouth disease. N Engl J Med.

[3] Ooi MH, Wong SC, Lewthwaite P, Cardosa MJ, Solomon T (2010). Clinical features, diagnosis, and management of enterovirus 71. Lancet Neurol.

[4] Chua KB, Kasri AR (2011). Hand foot and mouth disease due to enterovirus 71 in Malaysia. Virol Sin.

[5] Onozuka D, Hashizume M (2011). The influence of temperature and humidity on the incidence of hand, foot, and mouth disease in Japan. Sci Total Environ.

[6] Ooi E-E, Phoon M-C, Ishak B, Chan S-H (2002). Seroepidemiology of human enterovirus 71, Singapore. Emerg Infect Dis.

[7] Fujimoto T (2012). Hand, foot, and mouth disease caused by coxsackievirus A6, Japan, 2011. Emerg Infect Dis.

[8] Chang H-L (2012). The association between enterovirus 71 infections and meteorological parameters in Taiwan. PLoS One.

[9] Huang Y (2013). Effect of meteorological variables on the incidence of hand, foot, and mouth disease in children: a time-series analysis in Guangzhou, China. BMC Infect Dis.

[10] Wei J (2015). The Effect of Meteorological Variables on the Transmission of Hand, Foot and Mouth Disease in Four Major Cities of Shanxi Province, China: A Time Series Data Analysis (2009–2013). PLoS Negl Trop Dis.

[11] Xu M (2014). Non-Linear Association between Exposure to Ambient Temperature and Children’s Hand-Foot-and-Mouth Disease in Beijing, China. PLoS One.

[12] Zhu L (2015). The Impact of Ambient Temperature on Childhood HFMD Incidence in Inland and Coastal Area: A Two-City Study in Shandong Province, China. Int J Environ Res Public Health.

[13] Zheng X-y (2015). Association between air pollutants and asthma emergency room visits and hospital admissions in time series studies: a systematic review and meta-analysis. PLoS One.

[14] Barnett AG (2005). Air pollution and child respiratory health: a case-crossover study in Australia and New Zealand. Am J Respir Crit Care Med.

[15] Huang R, Bian G, He T, Chen L, Xu G (2016). Effects of meteorological parameters and PM10 on the incidence of hand, foot, and mouth disease in children in China. Int J Environ Res Public Health.

[16] X, L., L, Z., W, Z. & X, X. Correlation Analysis of 2010–2011 in Guangzhou City,Yuexiu District hand foot and mouth disease incidence and meteorological factors and air pollution index. *China Journal of Pharmaceutical Economic*, 182–184 (2013).

[17] Ning G (2018). Characteristics of air pollution in different zones of Sichuan Basin, China. Sci Total Environ.

[18] Yearbook, C. S. National Bureau of statistics of China. *China Statistical Yearbook* (2014).

[19] Mao Q (2014). Coxsackievirus A16: epidemiology, diagnosis, and vaccine. Hum Vaccin Immunother.

[20] Yip, C. C., Lau, S. K., Woo, P. C. & Yuen, K.-Y. Human enterovirus 71 epidemics: what’s next? *Emerg Health Threats J* **6** (2013). 10.3402/ehtj.v6i0.19780 PMC3772321 24119538

[21] Yu G (2019). Short-term effects of meteorological factors and air pollution on childhood hand-foot-mouth disease in Guilin, China. Sci Total Environ.

[22] Huang, R. *et al*. Impact of PM 10 and meteorological factors on the incidence of hand, foot, and mouth disease in female children in Ningbo, China: a spatiotemporal and time-series study. *Environmental Science and Pollution Research*, 1–12 (2018). 10.1007/s11356-018-2619-5 29961907

[23] Zhang Y, Peng M, Yu C, Zhang L (2017). Burden of mortality and years of life lost due to ambient PM10 pollution in Wuhan, China. Environmental Pollution.

[24] The Ministry of Health of China. Hand, Foot and Mouth Disease Prevention and Control Guideline, China. (2009) Available at, http://www.gov.cn/gzdt/2009-06/04/content_1332078.htm (Accessed: 4th June 2017).

[25] Gasparrini A, Armstrong B, Kenward M (2010). Distributed lag non-linear models. Stat Med.

[26] Gasparrini A (2014). Modeling exposure-lag-response associations with distributed lag non‐linear models. Stat Med.

